# Antibacterial Activities of *Calpurnia aurea* against Selected Animal Pathogenic Bacterial Strains

**DOI:** 10.1155/2020/8840468

**Published:** 2020-11-17

**Authors:** Getachew Mulatu

**Affiliations:** Department Veterinary Laboratory Technology, College of Agriculture and Veterinary Sciences, Ambo University, P.O. Box 19, Ambo, Ethiopia

## Abstract

The study aimed to determine the phytochemicals and to assess the antibacterial activities of crude extracts of different parts of *Calpurnia aurea* against *Staphylococcus aureus*, *Streptococcus pyogenes*, *Listeria monocytogenes*, *Escherichia coli* O157 H:7, *Salmonella typhi*, and *Campylobacter jejuni.* The fresh and healthy leaves, barks, stems, and roots of the plant parts were collected, herbarium, dried, and grounded, and bioactive compounds were extracted by ethanol (99%) and water. Mass of crude extracts was determined by using the Whatman No. 1 filter paper and rotary evaporator. Major secondary metabolites were also screened using phytochemical screening tests. Antibacterial activities (inhibition zones, mm) and minimum inhibition concentrations (MIC) were evaluated using agar-well diffused methods and agar dilution methods, respectively. The antibiotics ciprofloxacin, amoxicillin, penicillin, and tetracycline were used as positive controls at concentrations of 0.1 mg/ml and 0.2 mg/ml, while distilled water was used as the negative control. All the crude extracts were tested triplet (3x) for antibacterial activities against selected bacterial strains with two different concentrations 25 and 50 mg/ml and analyzed to compare the mean ± standard deviation between triplets. The results revealed that ethanol extracts showed high crude mass extracts, antibacterial activities, and major secondary metabolites such as alkaloids, tennis, flavonoids, saponins, steroids, and phlobatannins compared with aqueous extracts. Among antibiotics used, penicillin showed resistance to *S. aureus* and *E. coli* O157 H:7. *C. jejuni* was found to be the most susceptible bacterium to ethanol extracts' leaves, barks, and stems with MIC 3.125 mg/ml, whereas *S. aureus* was the least susceptible to all crude extracts. The study provided the traditional and scientific basis of *Calpurnia aurea* used against some bacterial diseases.

## 1. Background

In Ethiopia, people have been using ethnomedicinal plants traditionally to treat both human and animal diseases. It is documented that approximately 80% of the Ethiopian population relies on traditional medicine to cure ailments [1] since plants are rich in a wide diversity of secondary metabolites that have been found to exhibit antimicrobial, antioxidant, anti-infectious, and antitumor activities [2]. Also, plants are the most naturally effective and cheapest sources of drugs [3]. Especially, ethnoveterinary medicine is a holistic interdisciplinary study of the local knowledge, sociocultural structures, and environment associated with animal healthcare and husbandry to use medicinal plants [4]. Natural herbivore animals consumed or grazed herbs which might have tremendous antimicrobial activities to improve their life quality and maintain their health [5]. Such traditional medicine is still being used in rural and urban areas, but it is more widely practiced in rural areas where there is limited modern health service. However, little work has been done to promote traditional medicine which can be commercially available for veterinary practitioners [6].

Among ethnomedicinal plants, *Calpurnia aurea* (local name chekka by Afan Oromo) is a yellow-flowered shrub that is widely distributed throughout tropical Africa and has several ethnomedical uses in Ethiopia [7]. *Calpurnia aurea* is used for the treatment of amoebic dysentery and diarrhea, killing head lice, tapeworm and ticks, stomach-ache, bowel, syphilis, leishmaniasis, trachoma, bladder disorders, tinea capitis, wound, scabies, and elephantiasis different swellings in humans and animals [8]. According to some reports, this plant has secondary metabolic compounds such as saponins, steroids, alkaloids, tannins, flavonoids, terpenoids, and phlorotannins which were extracted from leaves, barks, stems, and roots of the plant by different extraction fluids such as ethanol, methane, acetone, and water [7, 9–12]. The secondary metabolic compounds of this plant have antimicrobial activities against pathogenic bacterial strains such as *Escherichia coli, Staphylococcus aureus*, *Salmonella* spp*., Lactobacillus* spp*., Streptococcus* spp*., Bacillus subtilis, Klebsiella pneumonia, Pseudomonas aeruginosa, Shigella sonnie, Shigella dysenteriae, Vibrio cholerae* microbacterium, and others reported by [9, 13–15].

*Salmonella* is a rod-shaped Gram-negative bacterium belonging to the family Enterobacteriaceae and is the causative agent of salmonellosis, which is characterized by two major syndromes: systemic septicemia and enteritis. Also, it can cause diarrhea, abortion, arthritis, respiratory diseases, necrosis of extremities, and meningitis. *S. typhi* and *S. paratyphi* produce typhoid in people, *S. gallinarum* in poultry, *S. abortusovis* in sheep, *S. choleraesuis* in pigs, *S. dublin* in cattle, etc. [16–18].

*Campylobacter* spp. are spiral, microaerobic, and Gram-negative bacteria that cause gastroenteritis in people and animals, e.g., *C. jejuni* subsp. *jejuni* (enteritis and abortion), *C. coli, C. mucosalis* (porcine enteritis), *C. hyointestinalis* subsp. *hyointestinalis* (porcine and bovine enteritis), *C. sputorum* (abortions in sheep), and *C. fetus* subsp. *fetus* (isolated from intestinal tracts of sheep and cattle, sporadic abortions) [17, 19, 20].

Listeriosis is a sporadic bacterial infection that affects a wide range of animals, people, and birds [21]. *Listeria monocytogenes* is a small, motile, Gram-positive, nonspore-forming, extremely resistant, diphtheroid coccobacillus that grows under a wide temperature range of 4°–44°C. The uterus of all domestic animals (ruminants) is highly susceptible to infection with *L. monocytogenes* at all stages of pregnancy, and it causes placentitis, fetal infection and death, abortion, stillbirths, neonatal deaths, metritis, and possibly viable carriers [19].

*Escherichia coli* is a Gram-negative, rod-shaped bacterium normally found in the intestine of poultry and most other animals. Colibacillosis occurs as acute fatal septicemia or subacute pericarditis, air vasculitis, salpingitis, and peritonitis. It is a common disease of economic importance in poultry and animals worldwide. However, no single *E. coli* serogroup used as a bacterium can provide full protection against all of the serogroups that cause infections. Virulence factors include possession of large virulence plasmids and the ability to resist phagocytosis and serum killing, acquire iron in low iron conditions, and adhere to host structures [19, 22].

*Staphylococcus aureus* is most commonly isolated from staphylococcosis cases, which is a Gram-positive, catalase-positive, and coccoid bacterium that appears in grape-like clusters on stained smears, but species such as *S. hyicus* have also been reported as the causative agent of osteomyelitis in Turkey poultry. Economic losses may result from decreased weight gain, decreased egg production, lameness, mortality, and condemnation at slaughter [19, 23, 24].

*Streptococci* are nonmotile, Gram-positive, and catalase-negative coccoid bacteria that occur singly, in pairs, or as short chains when observed on stained smears. *Streptococcus* species commonly associated with disease in avian species include *S. pyogenes, S. zooepidemicus* (*S. gallinarum*), *S. bovis*, *S. dysgalactiae*, *S. gallinaceus*, and *S. mutans*. *Streptococci* have been associated with acute septicemia, acute mortality with lameness, inappetence, diarrhea, joint infections, cellulitis, osteomyelitis, and endocarditis [22, 25].

*Calpurnia aurea* is scattered all over the parts of the country, and a sizable number of it has been used for the general treatment of diseased animals. However, there is no scientific basis for the therapeutic actions of traditional *Calpurnia aurea* medicines which might serve as the source for the development of more effective drugs. So, the objective of the current study was to evaluate the antimicrobial activities and to determine phytochemicals of crude extracts of *Calpurnia aurea* against a selected isolate of animal pathogenic bacterial strains.

## 2. Materials and Methods

### 2.1. Description of the Study Area

The study was conducted in selected kebeles of Guder town, West Shewa Zone of Oromia Regional State, which was located at 126 km west of Addis Ababa. The climatic condition of the area was 23% highland, 60% midaltitude, and 17% low land. It has annual rainfall and temperature ranging from 800 to 1100 mm and 16 to 22 C, respectively. The rainfall was bimodal with the short rainy season from February to May and the long rainy season from June to September. The area was found at a longitude of 370 46′ to 38 east and latitude of 80 59′ to 90 20′ north, and the altitude range is from 1600 to 3192 meters above the sea. The mixed type of agricultural activities was the practice in the area. The total livestock population was cattle 145,410, shoats 56,349, equine 44,845, and poultry 60,845 [26], but livestock productivity was poor due to prevalent diseases, malnutrition, poor genetic makeup, and management constraints.

### 2.2. Collection of the Plant Material

Healthy and fully matured leaves, barks, stems, and roots of *Calpurnia aurea* used in this study were collected in plastic bags appropriately labeled from Guder town between September and October 2018. The collected plant parts were confirmed by plant taxonomists and authenticated (herbarium) in the Plant Science Laboratory of Ambo University Guder campus.

### 2.3. Preparation of Crude Extracts

The collected plant parts were separately washed using tap water followed by sterilized distilled water and cut into smaller sizes of about 1-2 cm long. The washed plant parts were then shade-dried at room temperature for 15 days, pounded using an electric grinder into a fine powder, and finally kept in a refrigerator (4°C) until use.

#### 2.3.1. Preparation of Aqueous Extracts

Crude plant part extracts were obtained by separately suspending 200 g of each plant material in 1000 ml distilled water to give 20% (w/v) in a 2000 ml conical flask. The resulting leaf, bark, stem, and root powder suspension was then shaken at 121 rpm for 24 hrs using a shaker to produce the required infusion. After filtering the infusion using a double-layer cheesecloth and Whatman No. 1 filter paper, the filtrates were centrifuged for 15 min at 6000 rpm. The supernatants of the extracts were then preserved in airtight bottles until further use in the refrigerator (4°C) [27].

#### 2.3.2. Preparation of Ethanol Extracts

Two hundred grams of air-dried powdered plant materials were placed in 1000 ml of ethanol kept in a conical flask and were shaken in a rotary shaker at 121 rpm for 24 hrs. The suspension was filtered with a double-layer muslin cloth and Whatman No. 1 filter paper. The resulting filtrate was concentrated under reduced pressure in a rotary evaporator at 30 to 40°C for 30 min. The gummy residue was further dried in a water bath until the solvent was removed. After solvent evaporated, the remaining crude extracts were diluted with 10 ml sterile distilled water and kept in an airtight bottle in the refrigerator until use [28, 29].

### 2.4. Determination of Yields of Crude Extracts and Major Secondary Metabolites

Two hundred grams of powdered plant materials were used to obtain crude extracts from each plant part. The percentage yield for each plant part was the amount of crude extract recovered in mass compared with the initial amount of powdered plant materials used. It was presented in percentage (%) and was determined for each extraction solvent used.

One gram of each powdered sample was separately added into 20 ml of distilled water in test tubes. Then, the mixtures were boiled in a water bath for 7 min and were filtered while hot using the filter paper into Erlenmeyer flasks. After cooling, 1 ml of the filtrate was diluted to 5 ml solution using distilled water, and then a few drops (2-3) of 10% ferric chloride were added to it. Formation of bluish-black or brownish-green precipitate indicated the presence of tannins [30].

Solidified extract (0.5 gm) was taken in separate test tubes and mixed with 20 ml of distilled water. The mixtures were boiled in a water bath for 10 min. After cooling, the mixture was separately filtered through a Whatman No. 1 filter paper. Thereafter, 2 ml of 1% aqueous hydrochloric acid was added to each mixture and shaken to develop red precipitate that indicates the presence of phlorotannins [31].

Two milliliters of chloroform and 10 drops of acetic acid were mixed in a test tube. Then, 0.5 ml of concentrated ethanolic extract was added to the test tube and mixed with the solvent. Next, 1.5 ml of concentrated sulfuric acid was added from the side of the test tube. The change of red color through blue to green indicated the presence of steroids (Liebermann–Burchard test) [32].

Five milliliters of each concentrated ethanolic extract was mixed with 2 ml of chloroform in separate test tubes, and then 2 ml of concentrated sulfuric acid was added carefully and shaken gently to form a layer. Reddish-brown coloration of the interphase confirmed positive results for the presence of terpenoids (Salkowski test) [33].

One gram of each powdered sample was taken in separate test tubes and mixed with 10 ml of distilled water. Then, the mixtures were boiled in a water bath for 10 min and were filtered while hot into a 50 ml Erlenmeyer flask. The formation which above 1 cm of foam or froth confirmed the presence of saponins in the filtrate [30].

Two milliliters of each of the concentrated ethanolic extract was added to test tubes. Then, 4 drops of 10% NaOH solution were added and heated in a water bath for 10 min. The intensity of yellow color which became colorless on the addition of 10 drops of 1% hydrochloric acid showed the presence of flavonoids [34].

1.5 ml of 1% HCl was added to 4.5 ml of each concentrated ethanolic extract in different test tubes. Each mixture was heated and shaken for 2 min in a water bath. It was then cooled and filtered. The resulting filtrate was tested with Dragendorff's reagent for the presence of alkaloids as described by Adachukwu et al. [34]. 1 ml of the filtrate was added to 0.4 ml of Dragendorff's reagent. The formation of cream yellow precipitate indicated the presence of alkaloids.

### 2.5. Isolated Animal Pathogenic Bacterial Strains

Three strains of Gram-positive bacteria that infect animals, namely, *Staphylococcus aureus*, *Streptococcus pyogenes*, and *Listeria monocytogenes*, and three strains of Gram-negative pathogenic bacteria, *Escherichia coli* O157 H:7, *Salmonella typhi*, and *Campylobacter jejuni*, were obtained from Ethiopian Public Health Institute (EPHI) in icebox and transport media.

All bacterial cultures were first grown on 5% sheep red blood agar plates at 37°C for 18–24 hrs before inoculation onto the nutrient agar. Four up to five colonies of bacteria were selected and transferred with a sterile inoculating loop to a liquid medium and incubated until adequate growth equivalent to McFarland 0.5 turbidity units (1.5 × 10^8^ CFU/ml) standard was obtained. The inocula of the respective bacteria were streaked onto Mueller-Hinton agar (MHA) plates using a sterile swab in such a way as to ensure thorough coverage of the plates, and a uniform thick lawn of growth was obtained following incubation. The inoculated plates were left at room temperature for 3–5 min to allow for any surface moisture to be absorbed before applying the extract. Wells of 6 mm in diameter were formed onto MHA plates using a sterile cork borer. The wells were filled with the test agents (50 *μ*l each), and the plates were then allowed to stay for 1-2 hrs at room temperature for proper diffusion. Finally, the plates were incubated at 37°C for 18–24 hrs, and the resulting diameters of zones of inhibition were measured using a sliding caliper [29].

### 2.6. Evaluation of the Crude Extracts

The six test pathogens were exposed to aqueous and ethanol extracts of different plant parts (leaves, barks, stems, and roots) by adaptation of the agar-well diffusion method. The growth media were prepared following standard procedures. After complete solidification of the media, separate cultures of each species of bacteria were spread aseptically onto each plate. Immediately following this procedure, small wells (each with 6 mm diameter) on each inoculated plate were prepared aseptically using a sterile cork borer, and extracts of varying concentrations (25 and 50 mg/ml) were added into the wells. Plates were incubated at 37°C for 24 hours [35]. Overall, cultured bacteria with zones of inhibition equal to or greater than 7 mm were considered susceptible to the tested extract [36].

Ciprofloxacin, amoxicillin, penicillin, and tetracycline were used as positive controls at concentrations of 0.1 mg/ml and 0.2 mg/ml, with equal amounts as those of the extracts (50 *μ*l), and sterile distilled water (50 *μ*l) was used as a negative control. All plates were with three replicates. Sizes of colony diameter were measured after 24 hrs of growth at 37°C [17].

### 2.7. Determination of Minimum Inhibitory Concentration (MIC)

The ethanol and aqueous extracts of the different plant parts that showed significant antimicrobial activity in the previous test were selected for the determination of MIC. The minimum inhibitory concentration of the crude extracts of the leaves, the barks, the stems, and the roots of *Calpurnia aurea* was determined by the agar dilution method. The growth media were first prepared in the usual fashion and sterilized by autoclaving. The sterilized media were allowed to cool to 50°C, and 18 ml of the molten agar was added to test tubes which contain 2 ml of different concentrations of the crude extract and the control. The concentrations of the extracts used in this test ranged from 1.25 to 6.25 ml. The plates were dried at room temperature. The suspensions of the respective pathogens having density adjusted to McFarland 0.5 turbidity units (1.5 × 10^8^ CFU/ml) were inoculated onto the series of agar plates using a standard loop. Three loops of the suspension were transferred into each plate and incubated at 37°C for 24 hrs. The lowest concentration which inhibited the growth of the respective organisms was taken as the MIC [16, 17].

### 2.8. Data Management and Analysis

All the experiments were carried out in quadrant. Zones of inhibition were analyzed using Statistical Package for Social Sciences, version 16.0 (SPSS, Chicago, IL, USA). Other data were subjected to *T*-test for comparison, while those recorded from disc diffusion tests were analyzed using one-way analysis of variance (ANOVA) with multiple comparison tests to compare the mean ± standard deviation parameter within and between groups which were considered statistically significant at *p* < 0.05.

## 3. Results

### 3.1. Yields of Crude Extracts

The crude extracts used in this experiment were obtained from the extraction of 200 g powders of *C. aurea* parts using ethanol and water as extracting solvents. As indicated in Table 1, the number of extracts ranged from 4.2% to 9.1%. Ethanol extract of the leaves gave the maximum yield (9.1%) followed by the ethanol extract of the barks (8.35%). The lowest yield was obtained from the aqueous extract of the roots (4.2%).

### 3.2. Screened Phytochemicals from Crude Extracts

Phytochemical (secondary metabolic chemicals) screening of the ethanol extract of the *C. aurea* leaf revealed the presence of terpenoids, alkaloids, tannins, flavonoids, and saponins. *C. aurea* bark also had alkaloids, saponins, flavonoids, and steroids. The phytochemicals of the stem and root of *C. aurea* were less compared to the leaf and bark by ethanol extracts. Overall, phytochemical crude extracts by aquatic extracts were very low compared to ethanol extracts as shown in Table 2.

### 3.3. Antimicrobial Activities of Crude Extracts: Agar-Well Diffusion Method

In this study, the antimicrobial activities of the ethanol and aqueous crude extracts of the leaves, barks, stems, and, roots of *C. aurea* were evaluated using the agar-well diffusion method. A total of 8 crude extracts (ethanol and aqueous) were prepared and screened for antimicrobial activities against the test pathogens. The antimicrobial activities of different extracts of *C. aurea* against the six pathogenic bacteria (S. *aureus*, *S. pyogenes*, *L. monocytogenes, E. coli* O157 H:7, *S. typhi*, and *C. jejuni*) are presented in Tables 3–6. The antibacterial activities of plant parts extracted by ethanol 50 ml/mg against all tested isolates were not significantly different from one another (*p* > 0.05). Standard antibiotics (ciprofloxacin, amoxicillin, penicillin, and tetracycline) were used as positive controls and caused significantly higher zone of inhibition against all tested clinical isolates by both extracts (*p* < 0.05) except penicillin against *E. coli* O157 H:7 and *S. aureus*. Sterile distilled water which was used as a negative control had no antibacterial activity against all tested isolates (Tables 3–6).

The ethanol and aqueous crude extracts of the leaf at concentrations of 25 and 50 mg/ml were evaluated for in vitro antibacterial activities against the test pathogens. The zones of inhibition of crude extracts were 1.60–6.90 mm and 9.63–19.67 mm for aqueous and ethanol extracts, respectively. Ethanol extracts from leaves have a large inhibition zone, especially on *L. monocytogenes*, as shown in Table 3.

The antibacterial activities of the stem crude extracts were tested on selected pathogenic bacteria which imply that the ethanol extract showed significant growth inhibition against tested bacterial species. As indicated in Table 4, the zones of inhibition of the ethanol and aqueous stem extracts were in the range of 6.33–16.77 mm and 1.13–7.80 mm, respectively.

Similarly, the bark extracts were also tested for their antibacterial properties against the test pathogens. As indicated in Table 5, the diameters of the zones of inhibition ranged from 0.97 to 5.23 mm for aqueous bark extracts and 8.07–13.5 mm for ethanol bark extracts.

The results of the in vitro assays of antibacterial activities of the root extracts on the test pathogens are shown in Table 6. The ethanol extracts of the roots had inhibitory activities ranging from 6.10 to 13.87 mm, and the aqueous extracts resulted in the zones of inhibition ranging from 0.80 to 4.10 mm.

### 3.4. Minimum Inhibitory Concentration (MIC) of the Crude Extracts

The minimum inhibitory concentration (MIC) assay was employed to evaluate the effectiveness of the extracts that showed significant antimicrobial activities in the previous tests. MIC was determined for extracts that showed diameter greater than or equal to 7 mm of the growth inhibition zone at 25 mg/ml. All pathogens were added to the diluted ethanol extracts of concentrations ranging from 3.125 mg/ml to 12.5 mg/ml. The results are shown in Table 7. The data revealed that the MIC of ethanolic extracts ranged from 3.125 mg/ml bark and root extracts for *C. jejuni.* Leaf ethanol extract also scores 3.125 mg/ml on *L. monocytogenes*. Generally, the bark ethanol extracts had the lowest MIC, and the highest was for the leaf and stem ethanol extracts.

## 4. Discussion

The results of crude extract yields clearly showed that the percentage yield of the crude extracts of different plant parts varied from solvent to solvent. This could be attributed to different polarities and extracting potential of the solvents. Ethanol might be dissolved polar and nonpolar substances. As Justine et al. [37] reported, most antimicrobial agents that have been identified from plants are soluble in organic solvents, and this reveals the better efficiency of ethanol as an extracting solvent than aqueous. Table 1 also approved that the percentage yields of the crude extracts using the same extracting solvent varied from one part of the plant to the other. So, in the current study, when different plant parts were compared for their yield, the leaf extracts gave the maximum yield and the root extract the least for both extracting solvents. This indicates that the bioactive ingredients are not found uniformly through the plant, and some plant parts tend to have more bioactive compounds [38].

The screening of secondary metabolic chemicals from parts of *C. aurea* revealed that the major secondary compound groups were found in different parts of the plant and solvent type (Table 2). This finding has concurred with the report of [8] alkaloids, cardiac glycosides, flavonoids, phenols, phytosteroids, saponins, terpenoids, and tannins from *C. aurea* parts by the ethanolic extract. The report of [39–41] indicated that alkaloid, tannin, flavonoid, and saponin were present, but terpenoid and steroid were absent in 80% methanol extract of *C. aurea* leaves. The preliminary phytochemical analysis of 70% ethanolic extracts from the *C. aurea* seeds showed the presence of tannins, flavonoids, terpenoids, saponins, steroids, glycosides, and alkaloids compounds [41]. The stem and bark hexane extract of *C. aurea* yielded the widely studied isoflavonoids and alkaloid-type phytochemicals [42].

The phytochemical screening and qualitative estimation of *C. aurea* seeds and leaves showed that the leaves were rich in flavones and polyphenols than the seeds, yet the seeds are rich in alkaloids and tannins than the leaves of *C. aurea* [43]. Adedapo et al. [9] in their investigations showed that *C. aurea* has resulted in the isolation of a series of alkaloids, phenolic compounds, flavonoids, flavanols, and proanthocyanins from leaves, barks, and roots. The present finding was also in line with the report of Dula and Zelalem [44] which showed that the C. *aurea* root extract contains cardiac glycosides, tannins, flavonoids, terpenoids, saponins, steroids, alkaloids, and phenolic compounds by using ethanol, chloroform, methanol, and n-hexane extracts. Even if a standardized extraction protocol has not been developed for herbal extracts, 20–95% of the ethanol-aqueous mixture is frequently used by the herbal medicine industry to prepare ethanolic extracts [11]. Therefore, ethanol is widely used to obtain crude extracts of phytochemicals from plant materials in the herbal medicine industry for medication purposes. Due to the variation in the composition of active compounds, a given plant may require different concentrations of ethanol to achieve the maximum recovery of bioactive components. Moreover, the variation of secondary compounds may also exist within species and breed mainly due to plant genotypes, developmental stages, biotic factors (natural enemies, competitors, or mutualists), soil type and ingredients, the season of collection, and geographical locations [12, 45].

The present study indicated that the antibacterial activity of the ethanol extracts of *C. aurea* is much higher than aquatic extracts comparable to that of standard antibiotics. According to Adedapo et al. [9], stem extract by methanol had antibacterial activities, but in the current study, leaf extracts by ethanol had high antibacterial activities; the difference in the finding might be due to types of extracts.

Kulthe et al. and Tahera et al. [46, 47] reported the antibacterial activity of *Calpurnia* leaf extracts against nine enteric pathogens tested including *E. coli* O157 H:7, *S. typhi*, and other species. Likewise, Romha et al. and Kalayou et al. [48, 49] reported the antibacterial activity of *Calpurnia* leaf extracts against *E. coli*, *S. typhi, S. pyogenes*, and *S. aureus* clinical isolates. *C. jejuni* shows MIC on the leaf, bark, and root, while *L. monocytogenes* shows on leaf (3.125 mg/ml) ethanol extracts of *C. aurea* which is in line with the finding of Elisha et al. [14]. As commonly known, the Gram-negative bacteria are more resistant than the Gram-positive ones [9]; however, the current study showed that 3 of the Gram-negative bacteria used in this study were sensitive to this extract even at a high MIC of 3.125 mg/ml. Among the positive controls, antibiotic treatment with penicillin showed resistance to *E. coli* O157 H:7 and *S. aureus* at 0.1 and 0.2 mg/ml. This suggests that the *C*. *aurea* ethanol extract compound might be used instead of penicillin treatment of these disease-causing pathogens.

## 5. Conclusion

The current study revealed that *Calpurnia aurea* had alkaloids, saponins, tannins, flavonoids, terpenoids, steroids, and phlorotannins phytochemicals by aquatic and ethanol extraction methods from the plant parts (leaf, bark, stem, and root). Ethanol extract had antimicrobial activities against selected animal pathogens such as *E. coli* O157 H:7*, S. aureus*, *S. typhi, S. pyogenes, S. aureus, L. monocytogenes*, and *C. jejuni.* Also, this study showed that *E. coli* O157 H:7 and *S. aureus* had resistance against penicillin.

## Figures and Tables

**Table 1:** The percentage of yields of crude extracts.

Plant parts	Extraction solvents	Crude mass (g)	Percentage of crude yields
Leaves	Ethanol	18.2	9.1
	Water	13.6	6.8
Barks	Ethanol	16.7	8.35
	Water	12.0	6
Stems	Ethanol	15.3	7.65
	Water	10.1	5.05
Roots	Ethanol	11.2	5.6
	Water	8.4	4.2

**Table 2:** Screening of major secondary metabolites from parts of *C. aurea*.

Phytochemicals	Ethanol extract of *C. aurea* parts				Aquatic extract of *C. aurea* parts			
	Leaf	Bark	Stem	Root	Leaf	Bark	Stem	Root
Alkaloids	+	+	−	−	+	+	−	+
Tannins	+	−	+	−	+	−	−	−
Flavonoids	+	+	−	+	+	+	−	−
Terpenoids	+	−	−	+	−	−	−	−
Saponins	+	+	+	−	+	−	−	+
Steroids	−	+		+	−	−	−	−
Phlobatannins	−	−	+	−	−	−	−	−

(+) indicates the presence of suspected phytochemicals. (−) indicates the absence of suspected phytochemicals.

**Table 3:** Antibacterial activity of crude extracts of the leaf and antibiotics against the test pathogens (mean ± SD, *n* = 3).

Tested pathogens	Zone of inhibition							
	Crude extract solvents from the leaf			Antibiotics				
	Conc. Ml (mg)	Water (mean ± SD) (mm)	Ethanol (mean ± SD) (mm)	Conc. Ml/Mg	Ciprofloxacin (mean ± SD) (mm)	Amoxicillin (mean ± SD) (mm)	Penicillin (mean ± SD) (mm)	Tetracycline (mean ± SD) (mm)
*S. aureus*	25	3.57 ± 0.89^Aa^	11.2 ± 1.08^Ab^	0.1	11.83 ± 0.40^Cc^	13.37 ± 0.31^Ad^	—	21.13 ± 1.07^Bf^
	50	6.4 ± 1.74^Ba^	15.17 ± 1.73^Eb^	0.2	11.93 ± 1.03^Cc^	20.1 ± 1.59^Cd^	—	28.63 ± 3.06^Df^
*S. pyogenes*	25	5.5 ± 0.47^Da^	17.83 ± 1.29^Fb^	0.1	29.13 ± 1.97^Fc^	15.7 ± 0.76^Bd^	12.6 ± 0.58^Ae^	11.47 ± 0.66^Aa^
	50	6.53 ± 0.97^Ca^	13.3 ± 1.30^Ab^	0.2	30.47 ± 0.88^Fc^	29.97 ± 1.71^Fc^	30.13 ± 0.83^Fe^	25.47 ± 4.35^Cf^
*L. monocytogenes*	25	3.07 ± 1.34^Aa^	19.67 ± 0.59^Fb^	0.1	19.6 ± 0.56^Ac^	17.83 ± 0.87 ^Bd^	16.7 ± 0.80^Be^	31.33 ± 1.61^Ff^
	50	5.6 ± 1.81^Da^	9.63 ± 0.74^Cb^	0.2	25.8 ± 1.44^Bc^	23.23 ± 4.33 ^Cd^	28.2 ± 2.08^Ee^	31.63 ± 0.66^Ff^
*E. coli* O157 H:7	25	4.07 ± 0.52^Aa^	11.47 ± 0.66^Bb^	0.1	17.3 ± 3.62^Ac^	13.03 ± 2.61^Ad^	—	25.53 ± 3.16^Cf^
	50	6.9 ± 1.68^Ca^	15.67 ± 0.74^Eb^	0.2	19.73 ± 0.94^Ac^	16.8 ± 0.78 ^Bd^	—	27.87 ± 0.53^Ef^
*S. typhi*	25	2.67 ± 0.96^Fa^	11.5 ± 1.96^Bb^	0.1	16.27 ± 1.19^Dc^	26.57 ± 2.36 ^Ec^	15.2 ± 0.70^Be^	23.77 ± 1.35^Cf^
	50	1.7 ± 0.89^Ea^	13.23 ± 0.96^Ab^	0.2	22.27 ± 1.14^Ec^	26.87 ± 1.62 ^Ec^	23.87 ± 3.72^Ce^	28.13 ± 3.62^Ef^
*C. jejuni*	25	3.1 ± 0.82^Aa^	10.1 ± 0.32^Cb^	0.1	29.03 ± 1.26^Fc^	19.97 ± 0.77^Cd^	23.1 ± 2.0^Ce^	15.67 ± 0.90^Af^
	50	4.1 ± 0.87 ^Ba^	12.23 ± 1.04^Bb^	0.2	31.37 ± 0.86^Fc^	23.6 ± 3.49 ^Cd^	25.6 ± 2.25^De^	18.2 ± 1.49^Bf^

*n* = number of experimental replicates; SD = standard deviation; means with the same letter (lower case) in the same row are not significantly different; means with the same letter (upper case) in the same column are not significantly different.

**Table 4:** Antibacterial activity of crude extracts of the stem and antibiotics against the test pathogens (mean ± SD, *n* = 3).

Tested pathogens	Zone of inhibition							
	Crude extract solvents from the stem			Antibiotics				
	Conc. Ml (mg)	Water (mean ± SD) (mm)	Ethanol (mean ± SD) (mm)	Conc. Ml/Mg	Ciprofloxacin (mean ± SD) (mm)	Amoxicillin (mean ± SD) (mm)	Penicillin (mean ± SD) (mm)	Tetracycline (mean ± SD) (mm)
*S. aureus*	25	1.67 ± 0.50^Aa^	6.33 ± 0.76^Ab^	0.1	11.83 ± 0.40^Ac^	13.37 ± 0.31^Ad^	—	21.13 ± 1.07^Cf^
	50	1.13 ± 0.82^Aa^	7.8 ± 1.38^Bb^	0.2	11.93 ± 1.03^Ac^	20.1 ± 1.59^Cd^	—	28.63 ± 3.06^Ef^
*S. pyogenes*	25	2.1 ± 1.16^Ba^	10.17 ± 2.44^Cb^	0.1	29.13 ± 1.97^Ec^	15.7 ± 0.76^Bd^	12.6 ± 0.58^Ae^	11.47 ± 0.66^Aa^
	50	5.8 ± 0.41^Fa^	15.87 ± 1.88^Db^	0.2	30.47 ± 0.88^Fc^	29.97 ± 1.71^Fd^	30.13 ± 0.83^Fe^	25.47 ± 4.35^Df^
*L. monocytogenes*	25	1.63 ± 0.58^Aa^	6.4 ± 0.97^Ab^	0.1	19.6 ± 0.56^Cc^	17.83 ± 0.87^Cd^	16.7 ± 0.80^Be^	31.33 ± 1.61^Ff^
	50	7.3 ± 0.39^Fa^	14.67 ± 0.68^Db^	0.2	25.8 ± 1.44^Dc^	23.23 ± 4.33^Dd^	28.2 ± 2.08^Ee^	31.63 ± 0.66^Ff^
*E. coli* O157 H:7	25	3.47 ± 0.76^Ca^	12.03 ± 1.97^Cb^	0.1	17.3 ± 3.62^Bc^	13.03 ± 2.61^Ad^	—	25.53 ± 3.16^Df^
	50	7.8 ± 1.55^Fa^	12.47 ± 0.75^Cb^	0.2	19.73 ± 0.94^Cc^	16.8 ± 0.78^Bd^	—	27.87 ± 0.53^Ef^
*S. typhi*	25	3.87 ± 1.42^Ea^	12.13 ± 0.69^Cb^	0.1	16.27 ± 1.19^Bc^	26.57 ± 2.36^Ed^	15.2 ± 0.70^Be^	23.77 ± 1.35^Cf^
	50	3.7 ± 0.76^Ea^	16.77 ± 1.27^Eb^	0.2	22.27 ± 1.14^Dc^	26.87 ± 1.62^Ed^	23.87 ± 3.72^Ce^	28.13 ± 3.62^Ef^
*C. jejuni*	25	1.47 ± 0.68^Aa^	11.5 ± 1.05^Cb^	0.1	29.03 ± 1.26^Ec^	19.97 ± 0.77^Cd^	23.1 ± 2.0^Ce^	15.67 ± 0.90^Ba^
	50	2.43 ± 0.75^Ba^	12.2 ± 2.19^Cb^	0.2	31.37 ± 0.86^Fc^	23.6 ± 3.49^Dd^	25.6 ± 2.25^De^	18.2 ± 1.49^Bf^

*n* = number of experimental replicates; SD = standard deviation; means with the same letter (lower case) in the same row are not significantly different; means with the same letter (upper case) in the same column are not significantly different.

**Table 5:** Antibacterial activity of crude extracts of the bark and antibiotics against the test pathogens (mean ± SD, *n* = 3).

Tested pathogens	Zone of inhibition							
	Crude extract solvents from the bark			Antibiotics				
	Conc. Ml (mg)	Water (mean ± SD) (mm)	Ethanol (mean ± SD) (mm)	Conc. Ml/Mg	Ciprofloxacin (mean ± SD) (mm)	Amoxicillin (mean ± SD) (mm)	Penicillin (mean ± SD) (mm)	Tetracycline (mean ± SD) (mm)
*S. aureus*	25	1.67 ± 0.52^Aa^	10.6 ± 0.93^Bb^	0.1	11.83 ± 0.40^Fb^	13.37 ± 0.31^Fb^	—	21.13 ± 1.07^Cf^
	50	2.3 ± 0.18^Ba^	10.83 ± 2.07^Bb^	0.2	11.93 ± 1.03^Fb^	20.1 ± 1.59^Dd^	—	28.63 ± 3.06^Ef^
*S. pyogenes*	25	0.97 ± 0.18^Ag^	10.2 ± 0.76^Bb^	0.1	29.13 ± 1.97^Bc^	15.7 ± 0.76^Ed^	12.6 ± 0.58^Aa^	11.47 ± 0.66^Ab^
	50	3.1 ± 0.24^Ca^	13.5 ± 0.99^Eb^	0.2	30.47 ± 0.88^Ac^	29.97 ± 1.71^Ad^	30.13 ± 0.83^Fe^	25.47 ± 4.35^Df^
*L. monocytogenes*	25	1.77 ± 0.22^Aa^	8.07 ± 0.49^Aa^	0.1	19.6 ± 0.56^Dc^	17.83 ± 0.87^Fd^	16.7 ± 0.80^Bc^	31.33 ± 1.61^Ff^
	50	2.57 ± 0.21^Ba^	9.97 ± 1.04^Ab^	0.2	25.8 ± 1.44^Cc^	23.23 ± 4.33^Cd^	28.2 ± 2.08^Ee^	31.63 ± 0.66^Ff^
*E. coli* O157 H:7	25	1.03 ± 0.18^Aa^	10.33 ± 0.94^Bb^	0.1	17.3 ± 3.62^Ec^	13.03 ± 2.61^Fd^	—	25.53 ± 3.16^Df^
	50	5.23 ± 0.77^Ea^	13.23 ± 3.24^Eb^	0.2	19.73 ± 0.94^Dc^	16.8 ± 0.78^Ed^	—	27.87 ± 0.53^Ef^
*S. typhi*	25	2.13 ± 0.60^Ba^	11.13 ± 1.97^Cb^	0.1	16.27 ± 1.19^Ec^	26.57 ± 2.36^Bd^	15.2 ± 0.70^Bc^	23.77 ± 1.35^Cf^
	50	4.2 ± 1.71^Da^	12.77 ± 3.63^Db^	0.2	22.27 ± 1.14^Cc^	26.87 ± 1.62^Bd^	23.87 ± 3.72^Cc^	28.13 ± 3.62^Ef^
*C. jejuni*	25	1.1 ± 0.27^Aa^	12.5 ± 0.50^Db^	0.1	29.03 ± 1.26^Bc^	19.97 ± 0.77^Dd^	23.1 ± 2.0^Cc^	15.67 ± 0.90^Bb^
	50	2.63 ± 0.85^Ba^	12.47 ± 1.43^Db^	0.2	31.37 ± 0.86^Ac^	23.6 ± 3.49^Cd^	25.6 ± 2.25^De^	18.2 ± 1.49^Bf^

*n* = number of experimental replicates; SD = standard deviation; means with the same letter (lower case) in the same row are not significantly different; means with the same letter (upper case) in the same column are not significantly different.

**Table 6:** Antibacterial activity of crude extracts of the root and antibiotics against the test pathogens (mean ± SD, *n* = 3).

Tested pathogens	Zone of inhibition							
	Crude extract solvents from the root			Antibiotics				
	Conc. Ml (mg)	Water (mean ± SD) (mm)	Ethanol (mean ± SD) (mm)	Conc. Ml/Mg	Ciprofloxacin (mean ± SD) (mm)	Amoxicillin (mean ± SD) (mm)	Penicillin (mean ± SD) (mm)	Tetracycline (mean ± SD) (mm)
*S. aureus*	25	1.23 ± 0.40^Aa^	6.37 ± 0.50^Aa^	0.1	11.83 ± 0.40^Ab^	13.37 ± 0.31^Ab^	—	21.13 ± 1.07^Cf^
	50	3.9 ± 1.32^Da^	7.7 ± 0.50^Bb^	0.2	11.93 ± 1.03^Ab^	20.1 ± 1.59^Cd^	—	28.63 ± 3.06^Ef^
*S. pyogenes*	25	3.87 ± 0.83^Da^	9.37 ± 0.74^Cb^	0.1	29.13 ± 1.97^Ec^	15.7 ± 0.76^Bd^	12.6 ± 0.58^Ab^	11.47 ± 0.66^Ab^
	50	4.1 ± 0.18^Ea^	11.73 ± 1.39^Db^	0.2	30.47 ± 0.88^Fc^	29.97 ± 1.71^Fd^	30.13 ± 0.83^Fe^	25.47 ± 4.35^Df^
*L. monocytogenes*	25	1.67 ± 0.44^Aa^	6.1 ± 0.47^Aa^	0.1	19.6 ± 0.56^Cc^	17.83 ± 0.87^Bd^	16.7 ± 0.80^Cd^	31.33 ± 1.61^Ff^
	50	1.7 ± 0.36^Aa^	9.5 ± 0.56^Cb^	0.2	25.8 ± 1.44^Dc^	23.23 ± 4.33^Dd^	28.2 ± 2.08^Ee^	31.63 ± 0.66^Ff^
*E. coli* O157 H:7	25	3.47 ± 0.49^Da^	11.87 ± 1.19^Db^	0.1	17.3 ± 3.62^Bc^	13.03 ± 2.61^Ab^	—	25.53 ± 3.16^Df^
	50	3.43 ± 0.49^Da^	13.87 ± 1.09^Fb^	0.2	19.73 ± 0.94^Cc^	16.8 ± 0.78^Bd^	—	27.87 ± 0.53^Ef^
*S. typhi*	25	0.8 ± 0.09^Bg^	9.53 ± 0.50^Cb^	0.1	16.27 ± 1.19^Bc^	26.57 ± 2.36^Ed^	15.2 ± 0.70^Cd^	23.77 ± 1.35^Cf^
	50	1.57 ± 0.36^Aa^	12.57 ± 1.18^Eb^	0.2	22.27 ± 1.14^Dc^	26.87 ± 1.62^Ed^	23.87 ± 3.72^Dc^	28.13 ± 3.62^Ef^
*C. jejuni*	25	1.27 ± 0.3^Aa^	11.13 ± 1.08^Db^	0.1	29.03 ± 1.26^Ec^	19.97 ± 0.77^Cd^	23.1 ± 2.0^De^	15.67 ± 0.90^Bf^
	50	2.63 ± 0.28^Ca^	9.37 ± 0.57^Cb^	0.2	31.37 ± 0.86^Fc^	23.6 ± 3.49^Dd^	25.6 ± 2.25^Ee^	18.2 ± 1.49^Bf^

*n* = number of experimental replicates; SD = standard deviation; means with the same letter (lower case) in the same row are not significantly different; means with the same letter (upper case) in the same column are not significantly different.

**Table 7:** Minimum inhibitory concentration (MIC) of the extracts of leaves, fruits, stems, and roots of *C. aurea* against bacterial test pathogens in mg/ml.

The MIC of the ethanolic extracts (mg/ml)				
Pathogen (strain)	Leaf ethanol	Bark ethanol	Stem ethanol	Root ethanol
*S. aureus*	12.5	6.25	12.5	12.5
*S. pyogenes*	6.25	6.25	12.5	6.25
*L. monocytogenes*	3.125	6.25	12.5	12.5
*E. coli* O157 H:7	12.5	6.25	6.25	6.25
*S. typhi*	12.5	6.25	6.25	6.25
*C. jejuni*	3.125	3.125	6.25	3.125

## Data Availability

All the data used to support the findings of this study are available from the author upon reasonable request.
